# Detection of persistent *Plasmodium* spp. infections in Ugandan children after artemether-lumefantrine treatment

**DOI:** 10.1017/S003118201400033X

**Published:** 2014-05-16

**Authors:** MARTHA BETSON, JOSÉ C. SOUSA-FIGUEIREDO, AARON ATUHAIRE, MOSES ARINAITWE, MOSES ADRIKO, GERALD MWESIGWA, JUMA NABONGE, NARCIS B. KABATEREINE, COLIN J. SUTHERLAND, J. RUSSELL STOTHARD

**Affiliations:** 1 Department of Production and Population Health, Royal Veterinary College, Hatfield, Hertfordshire, UK; 2 Faculty of Infectious and Tropical Diseases, London School of Hygiene and Tropical Medicine, London, UK; 3 Vector Control Division, Ministry of Health, Kampala, Uganda; 4 Vector Control Division, Mayuge District, Uganda; 5 Department of Parasitology, Liverpool School of Tropical Medicine, Liverpool, UK

**Keywords:** artemisinin combination therapy, malaria, rapid diagnostic test, Uganda, *Plasmodium falciparum*, *Plasmodium malariae*, *Plasmodium ovale*

## Abstract

During a longitudinal study investigating the dynamics of malaria in Ugandan lakeshore communities, a consistently high malaria prevalence was observed in young children despite regular treatment. To explore the short-term performance of artemether-lumefantrine (AL), a pilot investigation into parasite carriage after treatment(s) was conducted in Bukoba village. A total of 163 children (aged 2–7 years) with a positive blood film and rapid antigen test were treated with AL; only 8·7% of these had elevated axillary temperatures. On day 7 and then on day 17, 40 children (26·3%) and 33 (22·3%) were positive by microscopy, respectively. Real-time PCR analysis demonstrated that multi-species *Plasmodium* infections were common at baseline, with 41·1% of children positive for *Plasmodium falciparum*/*Plasmodium malariae*, 9·2% for *P. falciparum*/ *Plasmodium ovale* spp. and 8·0% for all three species. Moreover, on day 17, 39·9% of children infected with falciparum malaria at baseline were again positive for the same species, and 9·2% of those infected with *P. malariae* at baseline were positive for *P. malariae*. Here, chronic multi-species malaria infections persisted in children after AL treatment(s). Better point-of-care diagnostics for non-falciparum infections are needed, as well as further investigation of AL performance in asymptomatic individuals.

## INTRODUCTION

Malaria remains a substantive public health problem in Uganda, particularly in young children. Challenges to effective malaria control in this country include an extremely high transmission intensity with entomological inoculation rates greater than 100 per year in many areas, a weak health system with a fragile supply chain for front-line antimalarial treatments, inadequate case management with poor diagnostic tools in rural health centres and ineffective monitoring and evaluation of control interventions (Yeka *et al.* 2012). To reduce the burden of clinical disease, Uganda adopted a fixed dose artemisinin combination therapy (ACT) of artemether-lumefantrine (AL), as its first-line treatment for uncomplicated falciparum malaria in 2006 (Batwala *et al.* 2010) and a number of trials indicate that AL is highly efficacious and safe in this country (Bukirwa *et al.* 2006; Dorsey *et al.* 2007; Kamya *et al.* 2007; Yeka *et al.* 2008; Achan *et al.* 2009; Arinaitwe *et al.* 2009; Bassat *et al.* 2009; The Four Artemisinin-Based Combinations (4ABC) Study Group, 2011). Despite concerns about non-compliance i.e. non-completion of the 3-day course of antimalarial therapy, high levels of adherence to home-based AL treatment and very similar high cure rates were found in a trial comparing unsupervised and supervised treatment (Fogg *et al.* 2004; Piola *et al.* 2005).

Since ACTs are relatively expensive and to ensure correct clinical management of febrile illness, the World Health Organization (WHO) promotes a ‘test-and-treat’ strategy for malaria involving parasitological diagnosis rather than presumptive treatment based on clinical indicators (WHO, 2010*a*). The gold standard for malaria diagnosis remains light microscopy, however, this can be difficult to implement in rural clinical settings due to a shortage of equipment and trained staff. Many African countries, including Uganda, are now introducing rapid diagnostic tests (RDTs) for malaria diagnosis in local health centres and for community-based malaria management (Drakeley and Reyburn, 2009). However, as there are a plethora of RDTs for detection of falciparum and vivax malaria produced by a range of manufacturers, which vary in format and diagnostic performance, adoption of the ‘best’ test can be problematic (WHO, 2012). Common antigen targets for RDTs, histidine-rich protein 2 (HRP2; *Plasmodium falciparum*-specific) and lactate dehydrogenase (LDH; pan-*Plasmodium* or species-specific), display marked differences in their sensitivity, stability and dynamics of clearance from the blood after antimalarial treatment (Aydin-Schmidt *et al.* 2013; Nyunt *et al.* 2013).

Although *P. falciparum* remains the major cause of morbidity and mortality in sub-Saharan Africa, non-falciparum infections, such as *Plasmodium vivax, Plasmodium malariae* and *Plasmodium ovale* spp., are also present in humans and their clinical significance should not be overlooked (Collins and Jeffery, 2005, 2007; Mueller *et al.* 2007, 2009). *Plasmodium malariae* and *P. ovale* spp. infections, in particular, are often challenging to diagnose by microscopy due to low parasitaemias and difficulties in distinguishing from *P. falciparum*, particularly in cases of mixed infection (Mueller *et al.* 2007). In fact, PCR-based methods have revealed that *P. malariae* and *P. ovale* spp. are more prevalent in sub-Saharan Africa than previously thought based on microscopic diagnosis (Mueller *et al.* 2007, 2009; Bruce *et al.* 2008; Oguike *et al.* 2011; Proietti *et al.* 2011). Unfortunately these methodologies are not readily deployed in remote resource-poor settings and the RDTs tested to date generally show poor diagnostic performance in detection of *P. malariae* and *P. ovale* spp. infections in individuals (Maltha *et al.* 2013*a*, *b*). In non-immune travellers, the sensitivities of RDTs range from 21·4 to 85·7% for *P. malariae* detection and from 5·5 to 86·7% for *P. ovale* (Houzé *et al.* 2011; Maltha *et al.* 2013*b*). There has been very little reported on the diagnostic performance of RDTs for these malaria species in endemic settings, likely due to their low prevalence (as detected by microscopy) and the fact that they often exist as mixed infections with other malaria species.

In line with national treatment guidelines and to develop a strategy for treatment of schistosomiasis in under 5 s, a closed-cohort longitudinal study was initiated in 2009 investigating schistosomiasis and malaria in mothers and young children living in six communities in Uganda (the Schistosomiasis in Mothers and Infants or SIMI project) (Betson *et al.* 2010). Over the course of this project AL was provided to all RDT-proven malaria cases at the time of the survey(s). Although on-site treatment decisions were based on RDT results, the vast majority of RDT-positives were subsequently shown to be parasitaemic by microscopy and PCR (Sousa-Figueiredo *et al.* 2010). In addition, we observed a substantial number of cases of co-infection of *P. falciparum* with *P. malariae* and/or *P. ovale* spp. (Oguike *et al.* 2011). More importantly, we noticed that many children in these areas of intense malaria transmission required repeated AL treatment, up to five times within 18 months. At the end of this longitudinal study, we considered that a pilot study was needed to gather evidence on the effectiveness of AL against *P. falciparum* and non-falciparum malaria in this setting. Here we report on a short-term observational follow-up study of children using microscopy, RDTs and molecular detection of parasites.

## MATERIALS AND METHODS

### Study area, participants and sampling

The SIMI (Schistosomiasis in Mothers and Infants) community-based longitudinal study was conducted in six villages on the shores of Lake Albert and Lake Victoria in Uganda, commencing in May 2009 (Betson *et al.* 2010; Stothard *et al.* 2011, 2013). A total of 1856 mothers and children under 6 years were recruited at baseline and followed-up at 3, 6, 12 and 18 months (Lake Victoria only) later. During the SIMI study, malaria diagnosis was carried out using a combination of Paracheck-Pf^®^ RDTs (Orchid Biomedical Systems, Goa, India) or First Response RDTs (HRP-2 based; Premier Medical Corporation, NJ, USA), OptiMAL RDTs (Diamed GmBH, Switzerland) and microscopy on Giemsa-stained blood films as described (Sousa-Figueiredo *et al.* 2010), as well as real-time PCR (see below). Prevalence levels of malaria infection in all mothers and children recruited to the SIMI study are summarized in Table 1 with stratification by lake and survey timepoint.

**Table 1.** Prevalence of malaria at different survey time points in mothers and children participating in the SIMI study at Lake Albert and Lake Victoria

Lake	Survey	Mothers		Children		Both	
		Slide % (95% CI) [*n*/*N*]	RDT % (95% CI) [*n*/*N*]	Slide % (95% CI) [*n*/*N*]	RDT % (95% CI) [*n*/*N*]	Slide % (95% CI) [*n*/*N*]	RDT % (95% CI) [*n*/*N*]
Albert	Baseline	22·0 (17·6–26·8) [72/328]	17·6 (13·5–22·2) [56/319]	60·5 (56·4–64·6) [339/560]	64·7 (60·6–68·7) [356/550]	46·3 (43·0–49·6) [411/888]	47·4 (44·0–50·8) [412/869]
	6 months	24·5 (19·2–30·4) [60/245]	24·3 (19·0–30·2) [59/243]	75·2 (70·8–79·3) [310/412]	76·0 (71·6–80·1) [314/413]	56·3 (52·4–60·1) [370/657]	56·9 (53·0–60·7) [373/656]
	12 months	17·7 (13·0–23·3) [40/226]	14·0 (9·9–19·0) [34/243]	66·0 (60·9–70·9) [241/365]	68·0 (63·2–72·5) [274/403]	47·5 (43·5–51·7) [281/591]	47·7 (43·8–51·6) [308/646]
Victoria	Baseline	26·5 (21·8–31·7) [87/328]	26·7 (22·0–31·8) [88/330]	82·7 (79·5–85·5) [520/629]	86·5 (83·6–89·1) [551/637]	63·4 (60·3–66·5) [607/957]	66·1 (63·0–69·1) [639/967]
	6 months	23·7 (18·4–29·5) [57/241]	24·9 (19·6–30·9) [60/241]	72·4 (68·2–76·4) [352/486]	72·2 (68·0–76·2) [351/486]	56·3 (52·6–59·9) [409/727]	56·5 (52·8–60·2) [411/727]
	12 months	30·9 (24·9–37·5) [68/220]	28·9 (23·1–35·3) [65/225]	70·2 (65·4–74·7) [278/396]	82·7 (78·8–86·2) [359/434]	56·2 (52·1–60·1) [346/616]	64·3 (60·5–68·0) [424/659]
	18 months	29·8 (23·7–36·5) [62/208]	35·5 (29·1–86·2) [77/217]	82·6 (78·6–86·2) [338/409]	88·9 (85·5–91·7) [384/432]	64·8 (60·9–68·6) [400/617]	71·0 (67·4–74·5) [461/649]

Bukoba village, Mayuge District, showed the highest prevalence of falciparum and non-falciparum malaria in young children (see Table 2) and so was chosen as the site for the AL follow-up study, which was conducted in November and December 2010 (Betson *et al.* 2010; Stothard *et al.* 2011). A group of children from Bukoba village (*N* = 163) within the SIMI cohort (*N* = 188) were recruited to the study on the basis of a positive First Response RDT result and microscopy-confirmed malaria. Children were subsequently followed up 7 and 17 days after day 0 (day of initiation of treatment). Due to financial and logistical constraints, only individuals who were RDT-positive on day 17 (see below), who had been given oral quinine sulphate as an alternative exit treatment, were followed up on day 24. A blood smear archive was made on days 0, 7 and 17, and confirmatory microscopy was carried out 2 or 3 days after each sample was taken (Sousa-Figueiredo *et al.* 2010). Blood spots were collected onto Whatman^®^ 3MM filter paper at all timepoints for molecular analyses (see below). In addition, haemoglobin (Hb) levels were recorded for each child using a HemoCue spectrometer (HemoCue AB, Angelholm, Sweden) on days 0, 7 and 17.

**Table 2.** Prevalence of different malaria species in mothers and children at baseline

Lake	Village	Mothers				Children			
		*N*	*P. falciparum* % (95% CI)	*P. malariae* % (95% CI)	*P. ovale* spp. % (95% CI)	*N*	*P. falciparum* % (95% CI)	*P. malariae* % (95% CI)	*P. ovale* spp. % (95% CI)
Albert	Bugoigo	130	41·5 (33·40–50·5)	1·5 (0·2–5·4)	0 (0–2·8)	216	65·7 (59·0–72·0)	1·4 (0·3–4·0)	1·4 (0·3–4·0)
	Walukuba	120	26·1^a^ (18·4–34·9)	1·7 (0·2–5·9)	0 (0–3·0)	194	61·7^b^ (54·4–68·5)	5·2 (2·5–9·3)	0·5 (0·01–2·8)
	Piida	82	36·6 (26·2–48·0)	0 (0–4·4)	0 (0–4·4)	161	67·7 (59·9–74·8)	6·2 (3·2–10·6)	0 (0–4·4)
Victoria	Bugoto	134	42·5 (34·0–51·4)	1·5 (0·2–5·3)	0 (0–2·7)	258	79·51 (74·0–84·2)	8·9 (5·7–13·1)	1·6 (0·4–3·9)
	Bukoba	126	47·6 (38·7–56·7)	2·4 (0·5–6·8)	0·8 (0·02–4·3)	248	87·5 (82·7–91·3)	14·5 (10·4–19·5)	8·5 (5·3–12·7)
	Lwanika	70	24·2 (14·8–36·0)	0 (0–5·1)	0 (0–5·1)	133	79·7 (71·9–86·2)	3·8 (1·2–8·6)	3·8 (1·2–8·6)

a *N* = 119.

b *N* = 193.

### Treatment

**Fig. 1.**
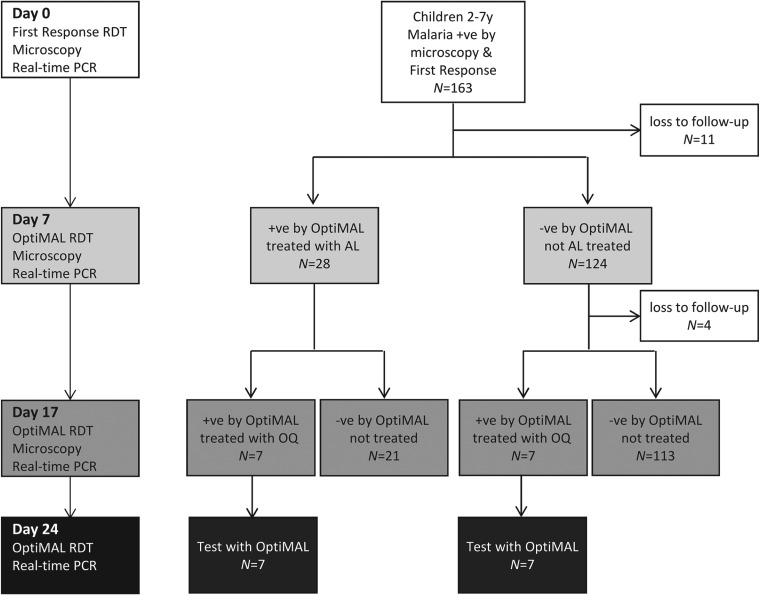
Schematic representation of the study protocol, showing tests employed at each time point, numbers of participants and treatment protocol. On days 7 and 17, treatment decisions were based on the results of OptiMAL tests (LDH) to allow rapid treatment in the field.

To allow rapid treatment in this village setting, treatment decisions were based on the results of RDTs (Fig. 1). At baseline the HRP2-based First Response Test was used, which demonstrates very high sensitivity in detection of *P. falciparum* infections (WHO, 2008). On Days 7 and 17, treatment decisions were based on the results of the OptiMAL RDT (Diamed GmBH, Switzerland), an LDH-based test, because the LDH antigen clears much more rapidly from the blood after treatment than HRP2, with average clearance times ranging from 3·7 to 7 days for LDH and 20 to 28 days for HRP2 (Aydin-Schmidt *et al.* 2013; Nyunt *et al.* 2013). On day 0 all participants were treated with AL (20 mg artemether/120 mg lumefantrine; LONART, Bliss Gvs Pharma Ltd., India) (WHO, 2010*a*). Two tablets of LONART were tested for quality and shown to contain exactly the expected amount of artemether and lumefantrine (H. Kaur, personal communication). A nurse administered the first AL dose, which was accompanied by fatty food, a mandazi (local doughnut). Subsequent AL doses were administered at home by the child's mother following the nurse's instructions. Children who were OptiMAL-positive on day 7 were treated a second time with AL. On day 17, OptiMAL-positive children were treated with oral quinine sulphate, the second-line antimalarial drug in Uganda (WHO, 2010*a*). At baseline children were tested for *Schistosomiasis mansoni* and soil-transmitted helminth infections by microscopic detection of eggs in stool as described (Katz *et al.* 1972; Betson *et al.* 2010). On day 0 all children were treated with praziquantel (40 mg kg^−1^) and albendazole (400 mg) according to WHO guidelines (WHO, 2006).

### Molecular analysis

To bolster microscopy findings and to detect non-falciparum infections, genomic DNA was extracted from dried blood spots using the chelex method (Dlamini *et al.* 2010) and real-time PCR diagnosis of *Plasmodium* infections was performed as described (Shokoples *et al.* 2009). A Ct value of 40 was used as a cut-off to differentiate between positives and negatives for each malaria species. To distinguish between recrudescent and new falciparum infections, genotyping of merozoite surface proteins (*msp1* and *msp2*) and glutamate-rich protein (*glurp*) was carried out according to published methods (Felger and Snounou, 2008). All PCR products were subject to polyacrylamide gel electrophoresis (10%) and fragment sizing was carried out using a GelDoc system and Image Lab gel analysis software (Biorad, Hercules, CA, USA). Infections were classified as either recrudescent or new infections on the basis of fragment sizes (WHO, 2007).

### Mapping of households

To build a comprehensive picture of spatial epidemiology of infections in the community, GPS coordinates of the households in the SIMI cohort have been collected (Stothard *et al.* 2011). The households of children in this study and their infection status by microscopy on days 7 and 17 were annotated using ArcView 9.3 (ESRI, CA, USA) GIS (Fig. 4). To determine whether there was any clustering of malaria infections on day 7 or day 17, the data were analysed using a Bernoulli model in SatScan v9.2.

### Statistical analysis

Epidemiological data were analysed using Stata v9.2 (StatCorp, TX, USA) and R v2.10.1 (The R Foundation for Statistical Computing, Vienna, Austria). Anaemia was categorized based on haemoglobin levels as follows: mild 10–11 g dL^−1^, moderate 7–10 g dL^−1^ and severe <7 g dL^−1^ (WHO, 2001). To determine how the different malaria species responded to AL treatment, the percentage of children infected with each species on days 7 and 17 (as assessed by real-time PCR) was calculated using the number of children infected with that species at baseline as the denominator (Fig. 3).

### Ethical approval

Ethical approval was provided by The London School of Hygiene and Tropical Medicine and the Ugandan National Council of Science and Technology. Informed consent was given by mothers on behalf of their children and documented in writing or by thumbprint (in cases of illiteracy).

## RESULTS

Table 3 details the characteristics of the AL follow-up study participants. The children ranged in age from 2–7 years and 48·5% were female. Only 8·7% of children had low-grade fever at baseline but 28·8% had moderate or severe anaemia. Although the prevalence of *S. mansoni* infection was very low, 9·0% of children had egg-patent hookworm infections.

**Table 3.** Characteristics of AL follow-up study participants (*N* = 163) and health indicators

Characteristic	Value
Age mean (range)	
Years	4 (2–7)
Gender n (%)	
Female	79 (48·5)
Clinical n (%)	
Axillary temperature >37·5 °C	14 (8·7)
Haemoglobin <10 g dL^−1^	47 (28·8)
Hepatomegaly^a^	25 (15·5)^b^
Splenomegaly^a^	68 (42·5)^c^
*S. mansoni* infection^d^	3 (1·9)^e^
Hookworm infection^d^	14 (9·0)^e^

a As recorded by physical palpation according to clinical guidelines.

b *N* = 160.

c *N* = 161.

d Egg patent upon faecal examination.

e *N* = 155.

**Fig. 2.**
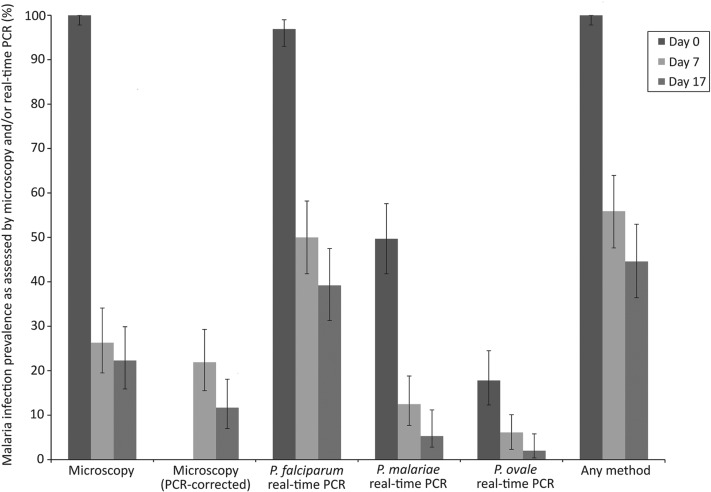
Malaria infection prevalence on days 0, 7 and 17 as assessed by microscopy, real-time PCR or either method. Error bars represent 95% confidence intervals.

Malaria infection status at different time points is summarized in Table 4 and Fig. 2. On day 0, all 163 children were positive by microscopy with a geometric mean parasitaemia of 1640·4 parasites *μ*L^−1^ blood. On day 7, 40 children (26·3%) remained malaria positive by microscopy and on day 17, 33 children (21·3%) had microscopically detectable malaria infections. After genotyping using *msp1, msp2* and *glurp* loci, 33 out of 40 and 17 out of 33 children were shown to have recrudescent *P. falciparum* infections on days 7 and 17, respectively. By real-time PCR 96·9% of children were positive for *P. falciparum* on day 0 and multi-species *Plasmodium* infections were common, with 41·1% positive for *P. falciparum*/*P. malariae*, 9·2% positive for *P. falciparum*/*P. ovale* spp. and 8·0% for all three species (Table 4; Fig. 2). Although there was a reduction in anaemia prevalence and severity over the course of the study, 22 children were anaemic on day 17 (Table 4).

**Table 4.** Parasitaemia and prevalence of malaria infection and anaemia at days 0, 7 and 17

		Day 0 *N* = 163 (95% CI)^a^ [*n*]	Day 7 *N* = 152 (95% CI) [*n*]	Day 17 *N* = 148 (95% CI) [*n*]
Parasitaemia (parasites *μ*L^−1^)	Geometric mean	1640·4 (1298·1–2073·0)	578·7 (351·0–954·2)	457·7 (185·2–1131·1)
	Range	20–90 640	31·4–10 360	5·4–101 080
Prevalence by microscopy (%)	Any parasitaemia	100 (97·8–100) [163]	26·3 (19·5–34·1) [40]	22·3 (15·9–29·9) [33]
	1–500 (parasites *μ*L^−1^)	18·4 (12·8–25·2) [30]	9·9 (5·6–15·8) [15]	10·1 (5·8–16·2) [15]
	500–2000 (parasites *μ*L^−1^)	37·4 (30·0–45·3) [61]	9·9 (5·6–15·8) [15]	8·1 (4·3–13·7) [12]
	2000–10 000 (parasites *μ*L^−1^)	33·1 (26·0–40·9) [ 54]	5·9 (2·7–10·9) [9]	0·7 (0·01–3·7) [1]
	>10 000 (parasites *μ*L^−1^)	11·1 (6·7–16·9) [18]	0·7 (0·02–3·6) [1]	3·4 (1·1–7·7) [5]
	PCR-corrected (*P. falciparum* only)	–	21·9 (15·5–29·3)^b^ [33]	11·7 (7·0–18·1)^c^ [17]
Prevalence by real-time PCR (%)	*P. falciparum*	96·9 (93·0–99·0) [158]	50·0 (41·8–58·2) [76]	39·2 (31·3–47·5) [58]
	*P. malariae*	49·7 (41·8–57·6) [81]	12·5 (7·7–18·8) [19]	6·1 (2·8–11·2) [9]
	*P. ovale* spp.	17·8 (12·3–24·5) [29]	5·3 (2·3–10·1) [8]	2·0 (0·4–5·8) [3]
Prevalence of mixed infections by real-time PCR (%)	*P. falciparum/P. malariae*	41·1 (33·5–49·1) [67]	10·5 (6·1–16·5) [16]	5·4 (2·4–10·4) [8]
	*P. falciparum/P. ovale* spp.	9·2 (5·2–14·7) [15]	3·3 (1·1–7·5) [5]	1·4 (0·2–4·8) [2]
	*P. falciparum/P. malariae/P. ovale* spp.	8·0 (4·3–13·3) [13]	1·3 (0·2–4·7) [2]	0·7 (0·01–3·7) [1]
Prevalence of anaemia (%)	Any anaemia	56·4 (48·5–64·2) [92]	45·4 (37·3–53·7) [69]	14·9 (9·6–21·6) [22]
	Mild	27·6 (20·1–35·1) [45]	27·6 (20·1–35·5) [42]	10·8 (6·3–17·0) [16]
	Moderate	27·6 (20·1–35·1) [45]	17·8 (12·0–24·8) [27]	4·1 (1·5–8·6) [6]
	Severe	1·2 (0·1–4·4) [2]	0	0

a 95% CI = 95% confidence interval.

b *N* = 151.

c *N* = 145.

**Fig. 3.**
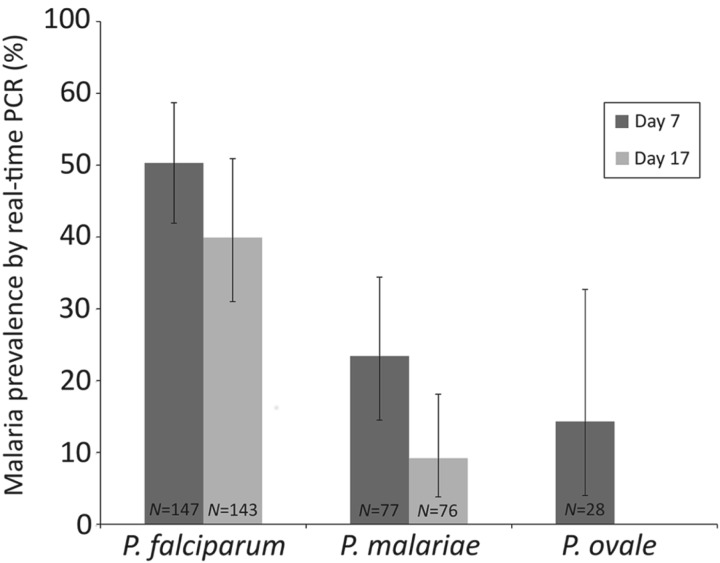
Percentage of the children positive for each malaria species at baseline (as assessed by real-time PCR) who were also positive on days 7 and 17. Error bars represent 95% confidence intervals. *N* = number of children in the denominator.

Table 5 summarizes malaria infection in relation to number of antimalarial treatments. Eleven of the 28 children who had received two consecutive treatments with AL (on days 0 and 7) were positive by microscopy on Day 17. At this timepoint, 16 children were *P. falciparum* positive by real-time PCR, six children were infected with *P. malariae* and two with *P. ovale* spp. On day 24, after two or three consecutive antimalarial treatments, 10 children were infected with *P. falciparum* and two were also infected with *P. malariae* (Table 5). When the performance of AL on different *Plasmodium* species was investigated, 39·9% of the children infected with falciparum malaria at baseline were still *P. falciparum* positive on Day 17, whereas 9·2% of those who were *P. malariae* positive at baseline showed *P. malariae* infection on Day 17 (Fig. 3).

**Table 5.** Summary of percentages of children who were malaria positive by PCR on days 17 and 24 after one or two antimalarial treatments

		Treatment(s)	Day 17		Day 24^a^	
			*N*	%	*N*	%
Microscopy		1 AL	120	18·3	NS	NS
		2 AL	28	39·3	NS	NS
Real-time PCR	*P. falciparum*	1 AL	120	35·0	7	57·1
		2 AL	28	57·1	7	85·7
	*P. malariae*	1 AL	120	2·5	7	0
		2 AL	28	21·4	7	28·6
	*P. ovale* spp.	1 AL	120	0·8	7	0
		2 AL	28	7·1	7	0

a All children who were followed up on day 24 had been treated with oral quinine on day 17.

1 AL = one AL treatment, 2 AL = two AL treatments; *N* = total number of individuals; NS = no slides.

**Fig. 4.**
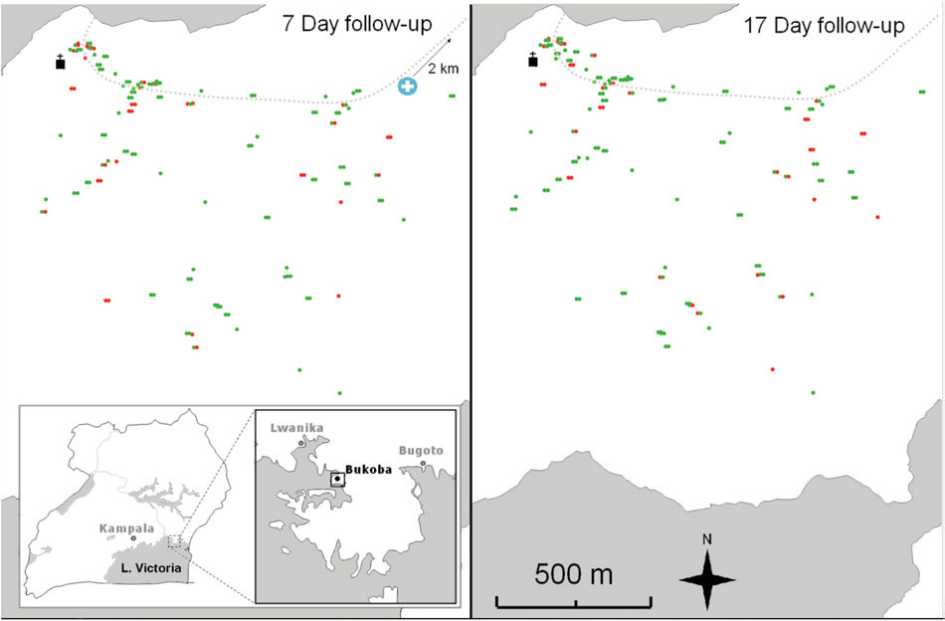
From left to right: map of positive (red) and negative (green) children according to microscopy at day 7; map of positive (red) and negative (green) children according to microscopy at day 17.

To determine whether there were differing risks of infection after treatment for children living in different parts of the village, their households and infection status on days 7 and 17 were mapped (Fig. 4). Spatial analysis using SatScan suggested six possible clusters of positive children on both day 7 and day 17. However, the statistical support for each cluster was low with *P* values greater than 0·1 in each case (data not shown). Thus strong evidence for clustering of malaria infections after AL treatment was not found, although this may reflect a lack of power due to the small numbers of infected individuals on days 7 and 17.

## DISCUSSION

In light of the challenges in diagnosis of falciparum and non-falciparum malaria and the chronically high prevalence of infections in Bukoba village, Uganda, we document the short-term effect of AL treatment on multiple malaria species in a cohort of young children living in this area. The study was designed to gain insights into treatment responses when AL was distributed to parasitaemic children in a community-based setting (unsupervised treatment), rather than as a carefully controlled standard efficacy trial. It is outside the remit of this paper to explore how well the results of standard efficacy trials predict the effectiveness of home-based management strategies in the same population. However, we found by microscopy that 26·3% of treated children (21·9% recrudescent by PCR genotyping) showed *P. falciparum* parasitaemia 7 days after treatment and that 22·3% (11·7% recrudescent) had parasites in their blood after 17 days, despite the fact that 33% of these positive children had already been retreated with AL at day 7. A substantial number of additional recurrent infections were identified by real-time PCR, including *P. malariae* and *P. ovale* spp. infections. Although none of these infections could be considered ‘complicated’ clinical malaria, their significance needs to be better quantified.

Our results are surprising given that clinical trials of AL in Uganda using microscopic diagnosis found that parasite clearance was rapid (very few detectable infections by day 3), there was negligible risk of parasitaemia before day 21 and that risks of treatment failure by day 28 ranged from 1–9% (Bukirwa *et al.* 2006; Dorsey *et al.* 2007; Kamya *et al.* 2007; Yeka *et al.* 2008; Achan *et al.* 2009; Arinaitwe *et al.* 2009; The Four Artemisinin-Based Combinations (4ABC) Study Group, 2011). In contrast, a trial in Ghana found day 28 failure rates of 14% (Owusu-Agyei *et al.* 2008) and a longitudinal community-based study in Papua New Guinea found a PCR-corrected parasitological treatment failure rate of 19·8% for AL (Schoepflin *et al.* 2011). Although artemisinin-tolerant falciparum has been reported in East Asia (Noedl *et al.* 2008; Dondorp *et al.* 2009), there has been little evidence to date of reduced efficacy of AL in Africa in standard clinical trials. Borrmann *et al.* reported a small decline in parasitological response rates to ACTs (including AL) in coastal Kenya (Borrmann *et al.* 2011). However, it is unclear whether this change reflected increased tolerance of the parasite to ACTs or reductions in clinical immunity due to decreased malaria transmission. In a number of African countries there appears to be selection for 86N, 184F and 1246D alleles in the product of the *P. falciparum* multidrug-resistant 1 gene (*pfmdr*1) in recurrent infections soon after AL treatment (Humphreys *et al.* 2007; Baliraine and Rosenthal, 2011; Gadalla *et al.* 2011). These polymorphisms as well as *pfmdr1* amplification have been associated with decreased sensitivity of *P. falciparum in vitro* to AL (Price *et al.* 2006). Thus, there is potential for tolerance to AL to develop in Africa.

This study was based on assessment of parasitaemia rather than clinical illness and most children had asymptomatic infections on day 0. The 21-day efficacy of the ACT dihydroartemisinin piperiquine (DP) for treatment of *P. falciparum* in parasitaemic but asymptomatic Ghanaian schoolchildren was 91% and it was suggested that chronic asymptomatic infections may not respond as well to antimalarial treatment as symptomatic infections because the parasites do not stimulate immune responses which can assist in parasite clearance (Dinko *et al.* 2013). In our study the performance was much lower, but there was no significant difference in the treatment responses of children with higher parasitaemia (>2000 parasites *μ*L^−1^) and those with lower parasitaemia (data not shown). Similar responses to AL were also observed when children with fever or moderate/severe anaemia on day 0 were compared with those with no fever or anaemia. However, the number of symptomatic individuals in our study may be too small to detect subtle differences in responses to AL treatment between symptomatic and asymptomatic individuals.

There has been little work assessing the dynamics of parasite clearance after AL treatment using PCR, although a median clearance time of 2 days has been reported (Aydin-Schmidt *et al.* 2013). However, additional submicroscopic infections have been detected in children during post-treatment follow-up using PCR (Gadalla *et al.* 2011; Aydin-Schmidt *et al.* 2013; Beshir *et al.* 2013; Dinko *et al.* 2013), as we report here. The importance of such sub-patent malaria infections was recently demonstrated by Beshir *et al.* in Kenya, who showed that residual sub-microscopic parasitaemia on day 3 after commencement of AL or DP treatment was associated with longer gametocyte carriage, increased transmission to mosquitoes and recurrence of microscopically detectable malaria infections on day 24 or day 48 (Beshir *et al.* 2013). The incorporation of PCR-based malaria diagnosis into the standard protocols of ACT efficacy studies will be important to determine the extent of submicroscopic carriage of malaria parasites after treatment.

The vast majority of *P. malariae* and *P. ovale* infections detected by real-time PCR at baseline were mixed infections with *P. falciparum*, which were difficult to detect on thick films despite cross-checking. *Plasmodium malariae* was not fully sensitive to AL in this study as around 9% of infected individuals were still positive on day 17, and two children were *P. malariae* positive at day 24 after three antimalarial treatments. The fact that 50% of study participants were infected with *P. malariae* and 18% with *P. ovale* indicates that the number of mixed infections receiving ACTs is substantially larger than previously thought. Mombo-Ngoma *et al.* reported, in 38 Gabonese patients, a 28-day cure rate by microscopy of 100% for AL in treatment of *P. malariae, P. ovale* and mixed infections in Gabon, although *post hoc* PCR revealed that only 19 of the patients in the study had been correctly diagnosed with non-falciparum malaria (Mombo-Ngoma *et al.* 2012). In contrast, Dinko *et al.* detected persistent *P. malariae* and *P. ovale* spp. infections in Ghanaian schoolchildren 21 days after ACT treatment using qPCR as the endpoint measure (Dinko *et al.* 2013). Therefore we strongly advocate that further studies are needed to understand the response of non-falciparum malaria to ACT treatment, and these should deploy molecular parasite detection as the primary endpoint. The development of specific molecular diagnostic tests for field-based detection of *P. malariae* and *P. ovale* spp. would be of great assistance in these endeavours.

It must be borne in mind that our study did not formally determine how well the participants complied with the approved antimalarial treatment regime, i.e. two doses a day for 3 days after consumption of fatty food (WHO, 2010*a*). Measurement of day 7 lumefantrine levels in our participants would have provided a means to verify adequate dosing. Thus, poor compliance may at least partially explain why some of the children remained parasitaemic after two or three doses of antimalarial medication. It must also be noted that de-worming drugs, praziquantel and albendazole, were co-administered with AL. There is a paucity of information on the putative interactions of anthelmintics with antimalarial drugs, and more research on this topic is warranted, especially given the changing landscape of mass drug administration in sub-Saharan Africa (WHO, 2010*b*). There are additional limitations to the analysis presented here. First, due to logistical constraints, study participants were only followed up for 17 or 24 days. It would have been preferable to extend this period to 28 or 42 days after treatment to allow a more direct comparison with published efficacy studies. Second, the real-time PCR analysis did not distinguish between asexual parasites and gametocytes. Although no gametocytes were observed by microscopy after AL treatment (data not shown), it is possible that sub-microscopic gametocytes were present. Third, it is possible that the proportion of new infections on day 7 and day 17 as determined by PCR genotyping is overestimated. Alleles can remain undetected using the recommended genotyping procedure particularly if they represent low abundance clones, leading to misclassification of recrudescences as new infections (Juliano *et al.* 2010). However, there is also the possibility of misclassifying new infections as recrudescences in areas of intense transmission, as children can be re-infected with parasites of the same genotype as the original infection. Thus care should be exercised when interpreting PCR ‘corrected’ results.

## CONCLUSION

The burden of malaria remains high in Uganda. Whilst the Ugandan malaria policy promotes AL as the first-line antimalarial treatment and is doubtless saving many lives, other challenges in its implementation remain. Our results suggest that even though ACTs remain highly efficacious in treatment of clinical disease in sub-Saharan Africa, asymptomatic and submicroscopic falciparum and non-falciparum infections often persist after treatment. These infections may be significant in terms of later recurrence of clinical disease and parasite transmission. Improved diagnostics are needed for field-based detection of non-falciparum malaria infections, gametocytes and drug-resistance markers. Increased community-based surveillance using a range of diagnostic tests is required to provide further insights into the extent of persistent parasitaemia after ACT treatment and for early detection of ACT-tolerant parasites in sub-Saharan Africa. Finally, new *in vivo* study protocols, with sensitive molecular endpoints, must now be developed that are specifically designed to measure the efficacy of antimalarial regimens against asymptomatic and sub-patent *Plasmodium* spp. infections.
